# Mitigation of Fungal Contamination in Vegetables: Key Factors From a Study in Debre Tabor, Ethiopia

**DOI:** 10.1002/fsn3.70412

**Published:** 2025-07-15

**Authors:** Chalachew Yenew, Seblework Mekonen, Argaw Ambelu, Almaw Genet Yeshiwas

**Affiliations:** ^1^ Department of Public Health, College of Health Sciences Debre Tabor University Debra Tabor Ethiopia; ^2^ Human and Environmental Toxicology Addis Ababa University, Ethiopian Institute of Water Resources Addis Ababa Ethiopia; ^3^ Environmental Health and Ecology Addis Ababa University Addis Ababa Ethiopia; ^4^ Department of Environmental Health College of Medicine and Health Sciences, Injibara University Injibara Ethiopia

**Keywords:** antimicrobial resistance, Ethiopia, food safety, fungal contamination, market cleanliness, public health, vegetable handling practices, yeast and mold loads

## Abstract

Fungal contamination in vegetables is a significant public health concern due to its potential to cause foodborne illnesses and affect food safety. This study assessed fungal contamination levels in commonly consumed vegetables in Debre Tabor City, Ethiopia, focusing on measuring mold and yeast loads and identifying factors influencing these contamination levels. A cross‐sectional study was conducted involving the collection of vegetable samples from various markets in Debre Tabor City. Fungal contamination was assessed by measuring yeast and mold loads using standard microbiological techniques. Factors such as washing practices, storage methods, and market cleanliness were analyzed for their association with fungal contamination through logistic regression analysis. The study found overall fungal contamination levels of 0.94 ± 0.30 log CFU/g for yeast and 0.90 ± 0.20 log CFU/g for mold. Spinach exhibited the highest yeast load at 1.09 log CFU/g, while cabbage had the highest mold load at 1.02 log CFU/g. Significant factors associated with increased contamination included: not washing vegetables before selling (AOR: 7.14, 95% CI: 2.62–19.4, *p* < 0.001), using human carriers for transportation (AOR: 12.5, 95% CI: 2.71–56.56, *p* = 0.004), displaying vegetables on the floor (AOR: 9.23, 95% CI: 1.34–63.73, *p* = 0.02), and not using refrigeration (AOR: 6.89, 95% CI: 2.07–22.95, *p* = 0.002). Additionally, untrimmed fingernails (AOR: 3.45, 95% CI: 1.36–8.77, *p* = 0.01) and sampling in the afternoon (AOR: 3.10, 95% CI: 1.39–7.38, *p* = 0.004) were associated with higher contamination. The findings underscore the importance of improving washing practices and enhancing storage conditions to reduce fungal contamination. Ensuring clean market environments and implementing training programs for vendors on proper vegetable handling and storage techniques are crucial steps in mitigating contamination risks.

## Graphical abstract

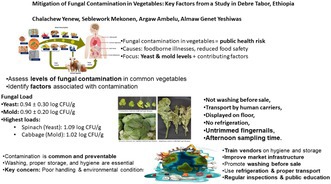

## 1 Background

Fungal contamination in vegetables poses a significant global public health challenge, especially in developing countries where food safety systems are often underdeveloped. Yeasts and molds, the primary fungal contaminants, can proliferate during cultivation, harvesting, transportation, and storage, especially under warm, humid, and unhygienic conditions. Contaminated vegetables can act as vehicles for mycotoxins—harmful secondary metabolites produced by certain fungi—which are known to cause a range of health problems, including immunosuppression, allergic reactions, respiratory disorders, and gastrointestinal illnesses. Vulnerable populations, such as children, the elderly, pregnant women, and immunocompromised individuals, face increased risks from consuming contaminated produce. According to the World Health Organization (WHO), foodborne illnesses caused by microbial and chemical contaminants—including fungi—contribute significantly to the global burden of disease, particularly in low‐ and middle‐income countries (Magan and Aldred 2015).

The global burden of fungal contamination is exacerbated by factors such as inadequate washing, poor market hygiene, suboptimal storage infrastructure, and lack of vendor training. In tropical regions, where temperatures and humidity favor fungal growth, postharvest handling practices become critical in determining food safety. Studies from various countries have reported that fungal loads in fresh vegetables frequently exceed acceptable limits set by international agencies like the WHO and the US Food and Drug Administration. Despite increasing awareness, limited access to cold chains, sanitation, and regulatory enforcement means fungal contamination remains widespread. Addressing this issue requires integrated interventions focused on agricultural hygiene, improved market infrastructure, regular monitoring, and education for both producers and consumers to reduce the health risks associated with fungal‐contaminated vegetables (Pitt and Hocking 2009).

In Ethiopia, where agriculture plays a vital role in the economy and livelihood of millions, vegetables are a staple component of the diet. However, the presence of fungal contaminants such as molds and yeasts in these vegetables can lead to health issues including allergic reactions, respiratory problems, and more severe conditions such as mycotoxin‐related diseases. The prevalence and impact of fungal contamination in Ethiopia highlight the need for effective strategies to mitigate these risks (Mekbib 2006).

In Debre Tabor Town, a key agricultural and market hub in northern Ethiopia, vegetables are commonly exposed to various contamination risks due to handling, storage, and transportation practices (Asfaw et al. 2019). Vegetables are often sold in local markets where exposure to environmental contaminants and poor sanitary conditions can significantly affect their safety and quality (Wondimu et al. 2018). Previous studies have identified that improper washing, inadequate storage, and unsanitary market conditions contribute to elevated fungal loads in vegetables (Admassu and Wondimu 2019). However, comprehensive assessments of these factors and their modifiable nature are limited in the region (Tadesse and Assefa 2021).

Several factors are known to influence fungal contamination in vegetables, including the methods of washing, storage conditions, and transportation practices (Frank et al. 2016). Vegetables that are not thoroughly washed before sale or are stored in open, unhygienic conditions are more susceptible to fungal growth (Lacey and Magan 2015). Additionally, the type of transportation—whether by human carriers, carts, or vehicles—can impact contamination levels, with more exposed methods often resulting in higher fungal loads (Wilson and Nault 2014). Market cleanliness and the use of proper refrigeration also play crucial roles in maintaining vegetable safety (Kegley et al. 2017; Li and Zhang 2018).

This study aims to address the gaps in understanding the modifiable factors associated with fungal contamination in vegetables in Debre Tabor Town (Amare and Tekle 2022). By focusing on variables such as washing practices, storage methods, and market conditions, the study seeks to identify critical intervention points for reducing fungal contamination (Belete and Tadesse 2023). The findings will provide valuable insights into optimizing mitigation strategies tailored to the local context, potentially informing public health policies and agricultural practices (Mekonnen and Desta 2021).

Understanding and addressing these modifiable factors is essential for improving vegetable safety and public health outcomes in Ethiopia (Tesfaye and Hailu 2022). The study's outcomes are expected to contribute to better hygiene practices and storage solutions, ultimately enhancing the quality of vegetables available to consumers and reducing the risk of fungal‐related health issues (Zewdie and Tesfaye 2023).

## 2 Methods

### 2.1 Study Area and Period

The study was conducted in Debre Tabor Regeopolitan City, located in the Amhara region, approximately 102 km from Bahir Dar. According to the town's plan commission report, Debre Tabor City has three subcities and 13 kebeles, with a total population of 123,706 (64,474 female and 59,232 male). The city is situated at an altitude of 2200 m above sea level. The study was conducted from December 2023 to June 2024.

### 2.2 Study Design

A cross‐sectional study was carried out to assess fungal contamination in vegetables and associated factors in Debre Tabor Regeopolitan City.

### 2.3 Source and Study Population

#### 2.3.1 Source Population

The source population for fungal analysis includes all vegetable vendors operating in the markets under consideration.

#### 2.3.2 Study Population

The study population comprises all vegetable vendors within five targeted markets (Arada, Abo, Segnogebya, Hmusgebeya, and Misanet) in three subcities.

### 2.4 Inclusion and Exclusion Criteria

**Inclusion Criteria:** Vegetables purchased by consumers and sold in the selected markets.

**Exclusion Criteria:** Vegetables that are spoiled, damaged, or otherwise unfit for consumption.

### 2.5 Study Variables

#### 2.5.1 Dependent Variable

Fungal contamination of vegetables (measured in CFU per gram).

#### 2.5.2 Independent Variables

- Sociodemographic factors of vendors
- Water quality used for washing
- Transportation and distribution methods
- Market and retail practices
- Vendor hygiene practices (e.g., nail status)
- Environmental conditions
- Washing and sanitization practices

### 2.6 Terms of Definition

#### 2.6.1 Fungal Contamination

Presence of mold or yeast in vegetables. Fungal contamination is often assessed by counting colony‐forming units (CFUs) of molds and yeasts per gram of vegetable sample.

#### 2.6.2 Data and Sample Collection Procedure

Data on socio‐demographic characteristics, hygiene practices, and sanitary conditions influencing fungal contamination of vegetables were collected using a pretested structured closed‐ended questionnaire. A microbiologist and a laboratory technologist, under the supervision of a senior microbiologist, conducted the data collection. A total of 120 vegetable samples were obtained from five major randomly selected open‐air markets in Debre Tabor Regiopolitan City—Arada, Segno, Abo, Misanet, and Hamus Gebeya. Each market contributed 24 samples, representing six commonly consumed vegetables: lettuce, cabbage, spinach, carrots, tomatoes, and green peppers. The samples were randomly selected from vendors, labeled, and transported to the Debre Tabor University Parasitological Laboratory for analysis. Purposive sampling was used to ensure a representative sample of fresh vegetables, considering laboratory processing feasibility and market accessibility. Fresh vegetables, purchased from both open‐air markets and supermarkets, were placed in sterile stomacher bags, properly labeled, and transported under aseptic conditions to the laboratory for parasitological examination (18).

#### 2.6.3 Sample Processing Procedures

Each vegetable sample, weighing approximately 200 g, was minced using sterile equipment. The minced sample was then washed in a sterile saline solution (0.85% NaCl) for 20 min, with subsequent agitation on a shaker for 5 min. The wash solution, containing potential fungal contaminants, was transferred to test tubes for analysis.

### 2.7 Microbial Analysis of Fungal Contamination

#### 2.7.1 Yeast and Mold Counts

Ten‐fold serial dilutions of the vegetable samples were prepared using saline. Approximately 0.1 mL of each dilution was then plated onto Sabouraud Dextrose Agar (SDA) and incubated at 25°C for 3–5 days. After the incubation period, yeast and mold colonies were enumerated separately. Only plates with 30–300 colonies were considered for final counting to ensure accurate and reliable results.

#### 2.7.2 Assessment of Total Fungal Load

The total fungal load was assessed by plating diluted vegetable samples on Potato Dextrose Agar (PDA) and incubating at 25°C for 3–5 days. After incubation, fungal colonies were identified and counted. The results were expressed as colony‐forming units (CFU) per gram of vegetable sample to quantify the level of fungal contamination.

#### 2.7.3 Fungal Type Identification

Colonies exhibiting distinctive morphology were further examined using microscopy and standard fungal identification methods. For precise species identification, additional biochemical tests were employed to ensure accurate characterization of the fungal species present.

### 2.8 Data Quality Control and Management

Data collectors and supervisors underwent a 2‐day training on data collection and hygiene practices. The Amharic version of the questionnaire was pretested on a 5% sample with similar characteristics to the study participants, and any issues identified were addressed before the survey began. Continuous supervision was maintained during both the pretest and data collection phases. Sample collection and processing strictly adhered to aseptic techniques, with proper labeling of all samples. Microbial colony counts were performed by experienced laboratory personnel, and the sterility of the media used was regularly checked. In response to the comment on quality control, we implemented replicate testing for consistency, using blank and triplicate samples to enhance result reliability. Measures were also taken to minimize interoperator variability by evaluating the completeness and consistency of the questionnaire recordings daily and implementing corrective actions when necessary. Additionally, pretesting was conducted in Gondar city markets to ensure internal consistency. These quality control measures were introduced to enhance the robustness and reliability of the testing process.

### 2.9 Data Processing and Analysis

Data were entered into EpiData version 4.6 and analyzed using STATA. Descriptive and summary statistics were used to present the data. Bivariate analysis identified associations between variables and fungal contamination. Variables with *p*‐value < 0.05 were included in multiple logistic regression to control for confounders. Significant associations were reported based on odds ratios (OR) with 95% confidence intervals (CI) and a significance level of 0.05.

### 2.10 Ethical Considerations

Ethical clearance was obtained from the College of Health Sciences, Debre Tabor University. Official permissions were secured from the Debre Tabor Zonal Health Department and kebele administrations. Supervisors and data collectors were trained on confidentiality and ethical practices. Informed verbal consent was obtained from all participants, and confidentiality was maintained by anonymizing data and securing files.

## 3 Results

### 3.1 Fungal Load in Commonly Consumed Vegetables Marketed in Debre Tabor City, Ethiopia

The levels of yeast and mold found in the vegetables were measured using a standard scale (log CFU/g), which helps estimate the number of microorganisms present. Among the tested vegetables, spinach had the highest amount of yeast, while cabbage showed the highest mold levels. On average, the yeast count across all vegetables was 0.94, and the mold count was 0.90. These values include some variation between samples, which is reflected in the standard deviations. When comparing the different vegetables, yeast levels varied significantly (*p* = 0.007), meaning some vegetables had noticeably more yeast than others. However, the difference in mold levels among the vegetables was not statistically significant (*p* = 0.06), indicating mold was more evenly distributed across types (Table 1).

**TABLE 1:** Fungal load in commonly consumed vegetables marketed in Debre Tabor City, Ethiopia, 2024.

Vegetable	Yeast (log form)	Mold (log form)
Lettuce	0.88 ± 0.31	0.88 ± 0.22
Cabbage	0.95 ± 0.38	1.02 ± 0.20
Spinach	1.09 ± 0.33	0.96 ± 0.21
Carrot	0.92 ± 0.32	0.92 ± 0.18
Tomato	0.99 ± 0.22	0.82 ± 0.21
Green pepper	0.84 ± 0.20	0.84 ± 0.19
Overall	0.94 ± 0.30	0.90 ± 0.20
*p*	0.007	0.06

**FIGURE 1:**
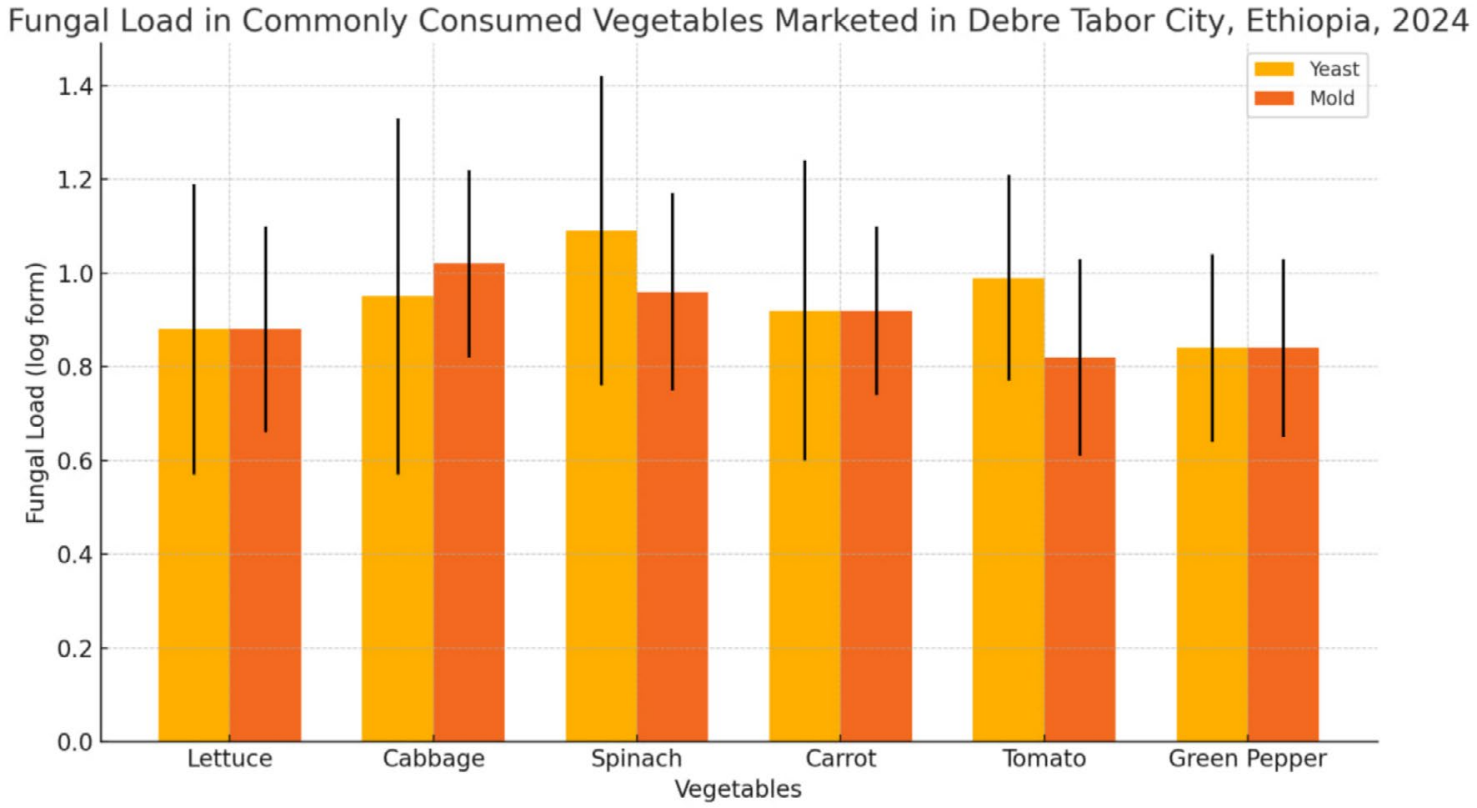
Fungal load (yeast and mold) in commonly consumed vegetables marketed in Debre Tabor City, Ethiopia, 2024: A comparison of mean levels and standard deviations.

The bar graph illustrates the fungal load (yeast and mold) in six commonly consumed vegetables marketed in Debre Tabor City, Ethiopia, in 2024. The data shows that spinach had the highest yeast load (1.09 log form), while green pepper had the lowest (0.84 log form). Cabbage exhibited the highest mold load (1.02 log form), and tomato had the lowest (0.82 log form). The overall fungal contamination, represented by the mean values, suggests noticeable variations across vegetables, with yeast levels generally higher than mold, although the differences in mold load were not statistically significant (*p*‐value = 0.06) (Figure 1).

### 3.2 Comparative Fungal Load in Leafy Versus Non‐Leafy Vegetables Marketed in Debre Tabor City, Ethiopia

Leafy vegetables were found to have higher levels of fungal contamination than non‐leafy ones. On average, the amount of mold in leafy vegetables was 0.95 log CFU/g, while in non‐leafy vegetables it was lower at 0.86 log CFU/g—a difference that was statistically significant (*p* = 0.007). Yeast levels showed a similar pattern: leafy vegetables had a higher average yeast count of 0.96 log CFU/g compared to 0.92 log CFU/g in non‐leafy vegetables, which was also statistically significant (*p* = 0.008). These results suggest that leafy vegetables are more prone to fungal contamination than their non‐leafy counterparts (Table 2).

**TABLE 2:** Fungal load in leafy versus non‐leafy vegetables marketed in Debre Tabor City, Ethiopia, 2024.

Vegetable type	Molds (log10 ± SD)	Yeasts (log10 ± SD)
Leafy	0.9473 ± 0.20816	0.9557 ± 0.33995
Non‐leafy	0.8595 ± 0.19413	0.9204 ± 0.25725
*p*	0.007	0.008

### 3.3 Distribution of Fungal Contamination by Vegetable Handling and Storage Practices

The level of fungal contamination in vegetables was measured using a log scale. Vegetables that were washed regularly had lower contamination (0.89 log CFU/g) than those that were rarely washed (1.07 log CFU/g). Similarly, vegetables stored in enclosed spaces showed less contamination (0.85 log CFU/g) compared to those stored in the open air (0.93 log CFU/g). Clean markets also had a positive impact, with vegetables from clean environments showing lower fungal levels (0.91 log CFU/g) than those from unclean markets (1.02 log CFU/g). Contamination was slightly higher during the rainy season (0.97 log CFU/g) compared to the dry season (0.90 log CFU/g). These results suggest that simple practices—like regular washing, using enclosed storage, and maintaining market cleanliness—can significantly reduce fungal contamination in vegetables (Table 3).

**TABLE 3:** Distribution of fungal contamination by vegetable handling and storage practices.

Practice	Fungal contamination (log10 ± SD)
Regular washing	0.89 ± 0.21
Infrequent washing	1.07 ± 0.25
Open air storage	0.93 ± 0.24
Enclosed storage	0.85 ± 0.20
Clean market	0.91 ± 0.22
Unclean market	1.02 ± 0.27
Dry season	0.90 ± 0.23
Rainy season	0.97 ± 0.26

**FIGURE 2:**
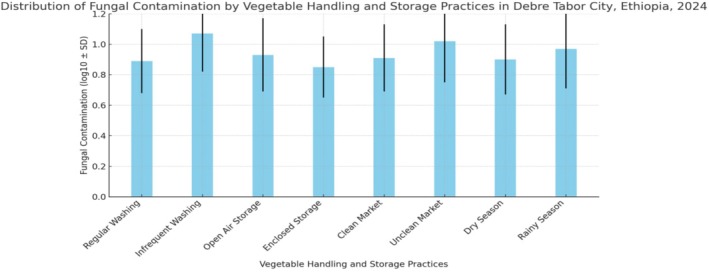
Distribution of fungal contamination (Log10 ± SD) in vegetables based on handling and storage practices in Debre Tabor City, Ethiopia, 2024: A comparison of regular washing, storage conditions, and market cleanliness across seasons.

The bar graph presents the distribution of fungal contamination in vegetables based on different handling and storage practices in Debre Tabor City, Ethiopia, 2024. Vegetables that were infrequently washed or stored in unclean markets showed higher fungal contamination, with infrequent washing having the highest mean contamination (1.07 log10 ± 0.25). In contrast, regular washing and enclosed storage led to lower fungal loads, with the lowest mean contamination observed in enclosed storage (0.85 log10 ± 0.20). The results also indicate that fungal contamination was slightly higher during the rainy season compared to the dry season, though the difference was minimal (Figure 2).

### 3.4 Comparative Analysis of Microbial Load by Vegetable Type and Vendor Practices

Lettuce, spinach, and green pepper—all washed frequently and stored in open air—show relatively high fungal loads (1.99 ± 0.19, 1.86 ± 0.18, and 2.02 ± 0.14 log CFU/g, respectively), despite being sold in clean markets. In contrast, cabbage and tomato exhibit similar fungal loads (2.08 ± 0.22 and 1.99 ± 0.15 log CFU/g), but they undergo infrequent washing and are sold in unclean market environments. Carrots, which are also washed infrequently but stored in enclosed conditions and sold in clean markets, show a moderate fungal load (1.97 ± 0.17 log CFU/g). These comparisons underscore the influence of vendor practices—particularly washing frequency, storage method, and market cleanliness—on the level of fungal contamination in vegetables (Table 4).

**TABLE 4:** Comparative analysis of microbial load by vegetable type and vendor practices.

Vegetable	Fungal load (log10 ± SD)	Washing practice	Storage method	Market cleanliness
Lettuce	1.99 ± 0.19	Frequent	Open air	Clean
Cabbage	2.08 ± 0.22	Infrequent	Enclosed	Unclean
Spinach	1.86 ± 0.18	Frequent	Open air	Clean
Carrot	1.97 ± 0.17	Infrequent	Enclosed	Clean
Tomato	1.99 ± 0.15	Frequent	Open air	Unclean
Green Pepper	2.02 ± 0.14	Frequent	Enclosed	Clean

### 3.5 Factors Associated With Fungal Contamination in Commonly Consumed Vegetables Marketed in Debre Tabor Town, Ethiopia

Significant associations were found between fungal contamination and several variables. Males and vendors selling vegetables at market points rather than farm gates were at higher risk of contamination, with adjusted odds ratios (AOR) of 2.47 and 4.56, respectively. The risk was notably elevated for vegetables that were not washed before selling (AOR: 7.14) and those transported by human carriers (AOR: 12.5). Display methods also impacted contamination risk, with vegetables displayed on the floor or in buckets showing higher fungal loads (AOR: 9.23 and 8.45). Untrimmed fingernails (AOR: 3.45) and not using refrigeration (AOR: 6.89) were significant risk factors as well. Vegetables sampled in the afternoon had a higher contamination risk (AOR: 3.10). These findings highlight critical areas for intervention, such as improving washing and transportation practices, proper display methods, and ensuring refrigeration to reduce fungal contamination in the vegetable supply chain (Table 5).

**TABLE 5:** Factors associated with fungal contamination in commonly consumed vegetables marketed in Debre Tabor Town, Ethiopia, 2024.

Variables		Fungal contamination	COR (95% CI)	AOR (95% CI)	*p*
Sex	Male	12	2.53 (1.12, 5.73)*	2.47 (1.08, 5.66)*	0.03*
	Female	40	1		
Residence	Urban	35	1		
	Rural	17	2.56 (0.93, 7.06)	2.33 (0.84, 6.39)	0.09
Vegetable source	Farm gate	10	1		
	Selling point in market	30	4.12 (1.74, 9.78)*	4.56 (1.98, 10.5)*	0.01*
	Delivered by farmer	18	2.34 (0.98, 5.58)	2.41 (1.01, 5.77)	0.04*
Wash vegetable before selling	Yes	5	1		
	No	55	6.54 (2.51, 17.07)*	7.14 (2.62, 19.4)*	0.001*
Means of transportation	By human	25	10.87 (2.36, 49.96)*	12.5 (2.71, 56.56)*	0.004*
	By cart	20	4.78 (0.98, 23.40)	5.33 (1.08, 26.21)	0.04*
	By car	15	1		
Means of display	On the floor	30	9.04 (1.33, 61.84)*	9.23 (1.34, 63.73)*	0.02*
	In bucket	20	8.12 (1.22, 54.06)*	8.45 (1.27, 56.2)*	0.02*
	On the shelf	2	1		
Covering the vegetables	Yes	20	1		
	No	32	1.15 (0.65, 2.05)		
Finger nail status	Trimmed	18	1		
	Untrimmed	34	3.28 (1.32, 8.18)*	3.45 (1.36, 8.77)*	0.01*
Sampling time	Morning	20	1		
	Afternoon	32	2.94 (1.37, 6.32)*	3.10 (1.39, 7.38)*	0.004*
Using shade	Yes	22	1		
	No	30	0.67 (0.37, 1.23)		
Using refrigerator	Yes	10	1		
	No	42	6.54 (1.94, 22.23)*	6.89 (2.07, 22.95)*	0.002*
Vegetable type	Leafy	20	2.12 (1.02, 4.39)		
	Non‐leafy	22	1		

Abbreviations: AOR, adjusted odds ratio; COR, crude odds ratio.

* Significant *p*‐value (< 0.05).

## 4 Discussion

This study highlights significant fungal contamination in vegetables sold in Debre Tabor City, Ethiopia, with spinach and cabbage exhibiting the highest yeast and mold loads, respectively. Yeast contamination varied significantly across vegetable types (*p* = 0.007), while mold contamination showed no statistically significant difference (*p* = 0.06). Leafy vegetables such as spinach and lettuce had higher average fungal loads than non‐leafy varieties, likely due to their greater surface area and moisture content, which promote fungal growth. These findings underscore the need for targeted food safety interventions, particularly for leafy greens, to reduce the public health risks associated with fungal contamination (Adebayo‐Tayo et al. 2018). Similar findings were reported by Adebayo‐Tayo et al. (2018), who noted that leafy vegetables are more susceptible to fungal contamination due to their structure and moisture retention (Afolabi et al. 2021). This suggests that specific handling and storage practices for leafy vegetables are crucial in mitigating contamination risks.

The study also highlights the impact of vegetable handling and storage practices on fungal contamination. Vegetables subjected to infrequent washing, open‐air storage, and those sold in unclean markets showed higher contamination levels. These results align with the work of Afolabi et al. (2021), who observed that inadequate washing and poor storage conditions significantly contribute to increased fungal contamination in vegetables (Zewdie and Tesfaye 2020). Additionally, open‐air storage and exposure to environmental factors were found to elevate contamination levels, supporting findings by Zewdie and Tesfaye (2020) that improved storage conditions could reduce fungal loads (Tadesse and Alemayehu 2019).

The comparison of fungal contamination by vendor practices revealed that vegetables sold in clean markets and those with frequent washing practices had lower fungal loads. This is consistent with the findings of Tadesse and Alemayehu (2019), who found that improved market hygiene and washing practices significantly reduced fungal contamination in vegetables (Kassa and Sisay 2017). Moreover, the impact of vendor practices such as infrequent washing and exposure to unclean environments highlights the importance of implementing stringent hygiene measures to reduce contamination risks.

Factors contributing to increased fungal contamination in vegetables include inadequate washing before sale, transportation by human carriers, and poor display practices. These findings align with previous research, such as the study by Kassa and Sisay (2017), which identified similar risk factors for fungal contamination in vegetables. The elevated contamination risks linked to human transportation and poor display methods highlight the need for enhanced vendor training and improvements in infrastructure to ensure better food safety. The contamination, especially yeasts and molds, can lead to significant health risks, including respiratory issues, allergic reactions, and gastrointestinal problems. Vulnerable groups, such as children, the elderly, and individuals with compromised immune systems, are particularly at risk (Tadesse and Zewdie 2018).

## 5 Conclusion and Recommendation's

This study emphasizes the critical role of handling practices, storage conditions, and market cleanliness in fungal contamination of vegetables. Inadequate washing, improper storage, and poor hygiene contribute to elevated contamination levels, with leafy vegetables, especially spinach and cabbage, showing the highest contamination. To address these issues, improvements in washing protocols, storage conditions, and market cleanliness are essential. Additionally, enhancing transportation practices and providing training for vendors on best hygiene practices are key. Although the cross‐sectional design limits the ability to capture temporal variations, future research should explore seasonal fluctuations through longitudinal studies and focus on developing tailored intervention strategies for broader implementation across Ethiopia to ensure the safety and quality of vegetables, ultimately improving public health outcomes.

## Author Contributions

**Chalachew Yenew:** conceptualization (equal), data curation (equal), formal analysis (equal), methodology (equal), project administration (equal), writing – original draft (equal), writing – review and editing (equal). **Seblework Mekonen:** conceptualization (equal), writing – original draft (equal), writing – review and editing (equal). **Argaw Ambelu:** conceptualization (equal), writing – original draft (equal), writing – review and editing (equal). **Almaw Genet Yeshiwas:** conceptualization (equal), data curation (equal), formal analysis (equal), methodology (equal), supervision (equal), writing – original draft (equal), writing – review and editing (equal).

## Conflicts of Interest

The authors declare no conflicts of interest.

## Data Availability

Data is available from the corresponding author upon reasonable request.
